# Increased galectin-3 may serve as a serologic signature of pre-rheumatoid arthritis while markers of synovitis and cartilage do not differ between early undifferentiated arthritis subsets

**DOI:** 10.1186/s13075-017-1282-4

**Published:** 2017-04-26

**Authors:** Saida Farah Issa, Anne Duer, Mikkel Østergaard, Kim Hørslev-Petersen, Merete L. Hetland, Michael Sejer Hansen, Kirsten Junker, Hanne M. Lindegaard, Jakob M. Møller, Peter Junker

**Affiliations:** 1 Department of Rheumatology, Odense University Hospital and Institute of Clinical Research, University of Southern Denmark, Odense, Denmark; 2 Department of Radiology, Copenhagen University Hospital at Herlev, Herlev, Denmark; 3 Copenhagen Center for Arthritis Research, Center for Rheumatology and Spine Diseases, Rigshospitalet, Glostrup, Denmark; 4 Department of Clinical Medicine, Faculty of Health and Medical Sciences, University of Copenhagen, Copenhagen, Denmark; 5 Research Unit at King Christian X Hospital for Rheumatic Diseases, Graasten, Denmark; 6 ReumaKlinik Roskilde, Støden 3, Roskilde, Denmark; 7 The Institute of Molecular Medicine, Cancer and Inflammation, University of Southern Denmark, Odense, Denmark

**Keywords:** Undifferentiated arthritis, Galectin-3, Collagen II, Hyaluronan, Cartilage degradation

## Abstract

**Background:**

Undifferentiated arthritis (UA) is a label applied to patients with joint complaints which cannot be classified according to current criteria, which implies a need for precision diagnostic technologies. We studied serum galectin-3, a proinflammatory mediator, and seromarkers of structural joint elements in patients with early, UA and their associations with disease profile and biochemical and imaging findings.

**Methods:**

One hundred and eleven UA patients were followed-up for at least 12 months and reclassified according to appropriate criteria (TUDAR). At baseline, demographics and laboratory and clinical disease measures, as well as wrist magnetic resonance imaging (MRI) synovitis, erosion, and bone marrow edema scorings, were recorded. Galectin-3, the type IIA collagen N-terminal propeptide (PIIANP), which is a marker of regenerative cartilage formation, and hyaluronan (HYA), which is prevalent in synovial tissue swellings, were measured by enzyme-linked immunosorbent assay (ELISA). Receiver operating characteristic (ROC) curve analysis was carried out to assess the discriminant capacity of galectin-3 against arthritis subsets.

**Results:**

Galectin-3 was increased in pre-rheumatoid arthritis (RA) (4.6 μg/l, interquartile range (IQR) 3.8–5.5) versus non-RA (4.0 μg/l, IQR 3.1–4.9; *p* = 0.03) and controls (3.8 μg/l, IQR 3.0–4.8; *p* = 0.009). PIIANP was equally depressed in either subset (*p* < 0.01). Galectin-3 in non-RA and HYA in UA did not differ from healthy controls. In the entire UA cohort, galectin-3 correlated with the MRI bone marrow edema score, while PIIANP correlated with the MRI erosion score, and HYA with the synovitis and erosion scores. ROC curve analysis showed that baseline galectin-3 discriminated well between pre-RA and non-RA with univariate area under the curve (AUC) of 0.64 (95% confidence interval (CI) 0.53–0.76) while AUC for galectin-3 + anti-CCP increased to 0.71 (95% CI 0.59–0.83).

**Conclusions:**

Galectin-3 in serum was increased in patients with early UA of pre-RA origin. Cartilage remodeling assessed by PIIANP was diminished in UA irrespective of subsequent clinical differentiation, while HYA did not differ from controls. ROC analysis showed a potential for galectin-3 to discriminate between pre-RA and non-RA.

**Trial registration:**

KF 11 315829. Registered 25 July 2006.

## Background

Undifferentiated arthritis (UA) is a diagnostic label which is used to categorize patients with arthritis complaints which cannot be classified according to current criteria [1]. In a review on inception cohorts of UA patients with monoarthritis or polyarthritis, in which arthritis was required to be present at inclusion, 17–32% progressed to rheumatoid arthritis (RA) after 1 year of follow-up [2]. Among UA patients with at least two swollen joints enrolled in the Norfolk Arthritis Register, 25–28% were in remission after 2 years [3, 4]. Although a number of risk factors for the development of RA have been identified, including female gender, smoking, socioeconomic status [5], and seropositivity for antibodies to cyclic citrullinated peptides (anti-CCP) [6] even in asymptomatic individuals [7], it is still not possible to predict with adequate certainty how patients with UA will ultimately segregate into distinctive disease entities. Several studies have investigated serological markers including acute phase reactants, e.g., C-reactive protein (CRP), erythrocyte sedimentation rate (ESR) [8], and inflammation modulators, e.g., matrix metalloproteinases (MMPs) [9], as potential predictors for RA development in UA. A number of prediction models for the clinical course of UA based on disease phenotype, serologic autoantibodies, and imaging data have been proposed in order to improve identification of UA patients at high risk for RA development [10, 11]. Recently, Duer-Jensen et al. reported that bone marrow edema of the wrist and metatarsophalangeal (MTP) joints detected by magnetic resonance imaging (MRI) is an independent predictor of future RA in patients with UA of short duration [12]. Combining MRI findings with clinical parameters and autoantibodies yielded additional discriminant capacity. In UA, there is a particular need to develop serological disease markers reflecting distinctive metabolic and inflammatory disease pathways to facilitate early diagnosis and to improve monitoring.

Several lines of evidence have implicated galectin-3 as a pro-inflammatory mediator in RA. Synovial inflammation and structural joint damage was lower in galectin-3^–/–^ mice with antigen-induced arthritis as compared with wild-type controls [13]. Ohshima et al. [14] reported that galectin-3 in the serum and synovial fluid was increased in patients with long-standing RA compared with osteoarthritis (OA) and controls. These authors also reported that galectin-3 mRNA and galectin-3 binding protein were expressed in particular at sites of cartilage and bone destruction in RA joints [14]. Moreover, they found that, although galectin-3 was only expressed in a few cells in OA synovial tissue, leucocyte infiltration was associated with upregulation of galectin-3 expression. In a 1-year prospective study on patients with newly diagnosed RA before treatment initiation, we recently reported that galectin-3 was increased, particularly in anti-CCP-positive smokers. Furthermore, galectin-3 was associated with bone marrow edema score of the wrist at baseline and remained elevated during 12 months of follow-up despite a targeted, synovitis-suppressive treatment strategy [15]. Conversely, we have previously reported that a seromarker of cartilage regeneration (type IIA collagen N-terminal propeptide (PIIANP)) is decreased in newly diagnosed RA [16–18] while hyaluronan (HYA), a marker of synovial swellings, is increased [19, 20].

Based on these observations, and the frequent occurrence of bone erosions in early RA before treatment [21, 22], we hypothesized that galectin-3 in the serum may be a pre-diagnostic signature of future RA. The aim of this investigation was to study the serum levels of galectin-3, PIIANP, and HYA in a cohort of patients with early UA (the TUDAR study [12]) and possible associations with disease profile, MRI findings, and molecular markers of cartilage remodeling and synovitis. In addition, we studied the capability of baseline galectin-3 to distinguish between pre-RA and non-RA according to diagnostic reassessment at 1 year follow-up.

## Methods

### Study population

**Fig. 1:**
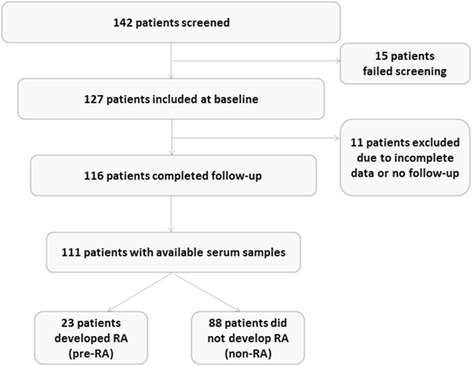
Flowchart showing patient disposition adapted from Duer-Jensen et al. [12]. *RA* rheumatoid arthritis

This study is based on patients with UA included in the TUDAR protocol [12] at their first visit to rheumatology outpatient clinics at Herlev Hospital (*n* = 21), Hvidovre Hospital (*n* = 46), or King Christian Xth Hospital for rheumatic diseases in Graasten (*n* = 44). Briefly, TUDAR is a prospective observational study on the capability of baseline MRI findings to predict future development of RA according to the 1987 classification criteria [23]. From June 2006 to July 2008, 127 patients who were at least 18 years old were included and followed for a minimum of 12 months (Fig. 1). Inclusion criteria were two or more tender and/or swollen joints among the metacarpophalangeal (MCP), proximal interphalangeal (PIP), wrist, or MTP joints persisting for 6–24 months. Further inclusion criteria were no contraindications for MRI and the absence of a specific rheumatic disease as assessed by a rheumatologist. Blood samples for biochemical and experimental laboratory analyses were drawn before initiation of drug treatment. Exclusion criteria were as follows: allergic reaction to MRI contrasts, pregnancy or breastfeeding, and treatment with intra-articular glucocorticoids before MRI examination and within 4 weeks before inclusion. At follow-up, patients were reclassified into RA according to the 1987 classification criteria or non-RA, whenever possible with the addition of a specific diagnostic label according to appropriate disease criteria.

### Control subjects

Self-reportedly healthy blood donors with an even distribution according to age (decades) and gender were recruited from the blood bank at Odense University Hospital as control subjects. One hundred and twenty subjects aged 20–65 years served as the reference population for galectin-3 and PIIANP, respectively, and 87 blood donors aged 23–64 for HYA.

### Clinical assessment

The following disease variables were recorded at inclusion and at follow-up: Health Assessment Questionnaire (HAQ), Disease Activity Score in 28 joints including CRP (DAS28-CRP), Visual Analogue Scales (VAS (0–100 mm) of pain, physician, and global assessment), a 40-joint count (tender or swollen joints), serum anti-CCP, and CRP.

### Biochemical analyses

Nonfasting blood samples were centrifuged at 3000 rpm for 10 min and were subsequently allowed to clot at room temperature. Following centrifugation, serum was stored at –80 °C until analysis. Serum samples from 111 of 116 patients with UA were available for analysis.

#### Galectin-3

Galectin-3 was measured using an enzyme-linked immunosorbent assay (ELISA; R&D systems, Minneapolis, USA) according to the manufacturer’s instructions. Galectin-3 standards, controls, and patient samples were dispensed into microplates coated with a mouse monoclonal antibody against Galectin-3. Upon incubation and washing, a polyclonal antibody against galectin-3 conjugated to horseradish peroxidase was added. Following washing to remove unbound antibody enzyme, a color reagent was added. The color development was halted and absorbance was measured within 30 min at 450 nm using an ELISA plate reader with the correction wavelength set at 540 nm. The intra- and interassay coefficient of variation in our laboratory amounted to 3.9% and 14.9%, respectively. The reference interval for serum galectin-3 was estimated to be 1.7–7.5 μg/l (2.5 and 97.5 percentiles) with a 95% confidence interval (CI) of 0.8–1.9 and 6.5–10.0 for the lower and upper limits, respectively.

#### PIIANP

PIIANP was quantified using ELISA (Millipore, Missouri, USA [24]) according to the manufacturer’s instructions. PIIANP standards, controls, and patient samples were added to the PIIANP antibody-coated wells in the presence of a competing biotinylated PIIANP peptide. Following incubation at room temperature for 2 h and washing, a streptavidin-horseradish peroxidase conjugate was added to the microplates. Upon adding a color substrate solution, absorbance was read within 5 min at 450 nm and 590, and the difference between absorbance units was recorded. The intra- and interassay coefficient of variation of PIIANP was 3.5% and 7.1%. The reference interval for serum PIIANP was 1327–4680 μg/l (2.5 and 97.5 percentiles) with a 95% CI of 894–1391 and 4234–4874 for the lower and upper limits, respectively.

#### HYA

HYA was measured by ELISA (Corgenix, Colorado, USA) according to the manufacturer’s instructions. The assay used microwells coated with a highly specific hyaluronic acid binding protein (HABP). HYA standards, controls, and patient samples were dispensed into the microwells. Upon incubation and washing, an enzyme-conjugated version of HABP was added to detect HYA in the samples. A color reagent was added and absorbance was measured within 30 min at 450 nm. The intra- and interassay coefficient of variation of HYA amounted to 3.6% and 6.1%, respectively. The reference interval for HYA was 2.4–77.0 μg/l (2.5 and 97.5 percentiles) with a 95% CI of 2.3–5.3 and 44.0–81.1 for the lower and upper limits, respectively.

#### Anti-CCP antibodies and serum CRP

Anti-CCP antibodies and serum CRP (mg/l) were measured according to standard laboratory procedures applied in the participating departments, with cut-off values of 5 U/ml and 5 mg/l, respectively.

### MRI

MRI of the dominant extremity (wrist, MCP, PIP, and MTP) was obtained before and after intravenous injection with gadolinium-based contrast (0.2 ml/kg; Dotarem, Guerbet, Roissy, France) within 2 weeks from inclusion. An MRI-experienced rheumatologist evaluated the images for RA pathologies according to the OMERACT (Outcome Measures in Rheumatology) MRI scoring system (RAMRIS) [25], while an experienced radiologist evaluated the images for other pathologies. All MRI evaluations were performed separately, and evaluators were blinded to clinical data. The MRI measures included evaluation of bone erosion score, synovitis score, and bone marrow edema score. Synovitis was scored from 0–3 in three different areas of the wrist and in each MCP, PIP, and MTP joint. Bone marrow edema was scored from 0–3 and bone erosion from 0–10 in each bone.

### Statistical analyses

Descriptive statistics concerning demographics, disease, and imaging variables for the groups are presented as medians and interquartile ranges (IQRs). The Mann-Whitney test was applied for comparison between groups. Univariate regression analyses were performed to assess correlations between galectin-3, PIIANP, or HYA and clinical and imaging variables. Subsequently, variables with a *p* value ≤0.20 were included in multiple regression analyses. Logistic regression was performed for the assessment of the predictive value of galectin-3 with regard to future RA development (diagnostic criteria fulfilled, yes/no) with adjustment for previously proposed risk factors, including age, gender, CRP, swollen joint count, tender joint count, anti-CCP [26], and bone marrow edema [12]. Finally, analysis of receiver operating characteristic (ROC) curves was performed in order to assess the capability of baseline galectin-3 to discriminate between pre-RA and non-RA in patients with UA at follow-up. *P* values ≤0.05 were considered statistically significant. The statistical analysis was performed using STATA version 13.

## Results

One hundred and sixteen patients participated after exclusion of patients with other rheumatic diseases at baseline, an incomplete dataset, or lack of follow-up. Among the 111 patients with available baseline sera, 23 were subsequently diagnosed with RA (pre-RA), while 88 had persistent UA, remitted, or had developed alternative classifiable conditions (non-RA), e.g., OA or psoriatic arthritis (Fig. 1). The median follow-up time was 17 months (12–23 months) [12]. At baseline, the pre-RA patients and non-RA group were comparable with regard to age and gender distribution and symptom duration. However, patients classified as having RA at follow-up had overall higher CRP (*p* < 0.01), more swollen (*p* = 0.03) and tender joints (*p* = 0.05), as well as higher VAS, HAQ, and DAS28-CRP scores at the time of inclusion (Table 1).

**Table 1:** Baseline demographic, clinical, and laboratory characteristics of patients with undifferentiated arthritis (UA)

Characteristic	Total UA (*n* = 111)	Pre-RA (*n* = 23)	Non-RA (*n* = 88)	*p* value (pre-RA vs non- RA)
Sex (M/F)	25/86	5/18	20/68	0.92
Age (years)	48 (41–57)	50 (44–59)	47 (39–56)	0.28
Anti-CCP (positive/negative)	15/96	8/15	7/81	**<0.001**
Symptom duration (months)	6 (4–12)	5 (4–12)	7 (4.5–12)	0.50
C-reactive protein (mg/l)	4 (3–8)	8 (4–14)	4 (3–6)	**<0.01**
Swollen Joint Count (40)	0 (0–2)	1 (0–2)	0 (0–2)	**0.03**
Tender Joint Count (40)	9 (5–20)	15 (5–27)	8 (4.5–17)	**0.05**
Visual Analogue Scale – Doctor (0–100 mm)	12 (5–24)	24 (18–30)	9 (3–19)	**<0.001**
Visual Analogue Scale – Pain (0–100 mm)	36 (17–54)	54 (21–69)	34 (17–51)	**0.01**
Visual Analogue Scale – Global (0–100 mm)	41 (20–59)	60 (40–71)	36 (15–55)	**<0.01**
DAS28-CRP	3.6 (2.9–4.3)	4.1 (3.6–5.1)	3.5 (2.7–4.2)	**<0.01**
HAQ score (0–3)	0.4 (0–1)	0.75 (0–1.3)	0.37 (0–0.75)	**0.05**
MRI wrist synovitis score	3 (2–5)	5 (3.5–5.5)	3 (2–5)	**<0.01**
MRI wrist erosion score	3 (1–5)	4 (2–5.5)	2 (0–4)	**0.01**
MRI wrist bone marrow edema score	0 (0–1)	1 (0–2)	0 (0–0)	**<0.01**

Results are presented as medians (interquartile range) unless otherwise stated

Significant *p* values are indicated in bold typeface

*Anti-CCP* antibodies to cyclic citrullinated peptides, *DAS28-CRP* Disease Activity Score in 28 joints including C-reactive peptide, *HAQ* Health Assessment Questionnaire, *MRI* magnetic resonance imaging, *RA* rheumatoid arthritis

When stratifying according to MRI findings in the two UA subgroups, pre-RA had higher wrist MRI synovitis scores (*p* < 0.01), bone marrow edema scores (*p* < 0.01) and erosion scores (*p* = 0.01). Regression analyses with either MRI MTP or wrist scores or MRI wrist + MTP scores yielded similar results. Accordingly, only MRI wrist data were included.

Outliers were identified based on *z* scores and included in the calculations. Concerning galectin-3, three outliers in the UA study population and two in the controls were identified. One PIIANP outlier was found in both the UA and control population. Similarly, two and five HYA outliers were identified in the UA and control population, respectively. Baseline serum levels of galectin-3, PIIANP, and HYA are presented in Table 2.

**Table 2:** Baseline levels of galectin-3, hyaluronan, and the type IIA collagen N-terminal propeptide

Marker	Reference interval	Total UA (*n* = 111)	Pre-RA (*n* = 23)	Non-RA (*n* = 88)	Controls (*n* = 120 or 87)	*p* value (pre-RA vs non-RA)	*p* value (pre-RA vs controls)	*p* value (non-RA vs controls)
Galectin-3 (μg/l)	1.65–7.54	4.1 (3.3–5.2)	4.6 (3.8–5.5)	4.0 (3.1–4.9)	3.8 (3.0–4.8)	**0.03**	**0.009**	0.56
Hyaluronan (μg/ml)	2.42–77.02	15.1 (8.5–30)	19.4 (8.5–30)	14.75 (8.6–30)	17.4 (10–25.8)	0.64	0.75	0.61
PIIANP (μg/l)	1327–4680	1800 (1439–2252)	1704 (1439–2105)	1801 (1436–2260)	2189 (1788–2712)	0.75	**0.004**	**0.0001**

Results are presented as medians (interquartile range) unless otherwise indicated

Significant *p* values are indicated in bold typeface

*PIIANP* type IIA collagen N-terminal propeptide, *RA* rheumatoid arthritis, *UA* undifferentiated arthritis

### Galectin-3

Galectin-3 was increased in the pre-RA subset (4.6 μg/l, IQR 3.8–5.5) compared with healthy controls (3.8 μg/l, IQR 3.0–4.8; *p* = 0.009) and non-RA (4.0 μg/l, IQR 3.1–4.9; *p* = 0.03).

When combining non-RA and pre-RA subsets, galectin-3 was slightly, but insignificantly, increased compared with healthy controls (*p* = 0.2). Galectin-3 in non-RA did not differ from healthy controls (*p* = 0.56). There was no significant difference in galectin-3 levels after stratification according to anti-CCP in the entire UA population and the pre-RA subset.

Univariate regression analyses including core disease measures and MRI findings showed that galectin-3 correlated positively with CRP, VAS pain, and MRI bone marrow edema. However, in the multiple regression model applied to the entire UA study population, CRP, VAS pain, and MRI bone marrow edema came out as nonsignificant.

Only imaging variables were included in regression analyses of pre-RA and non-RA subsets due to the small population samples after stratification. Following univariate regression analysis, galectin-3 in pre-RA did not correlate with MRI synovitis score, bone marrow edema score, or erosion score. Conversely, in non-RA, galectin-3 correlated positively with MRI bone marrow edema score (*p* = 0.02), also after adjustment for age and gender (*p* = 0.05).

### The type IIA collagen N-terminal propeptide

**Fig. 2:**
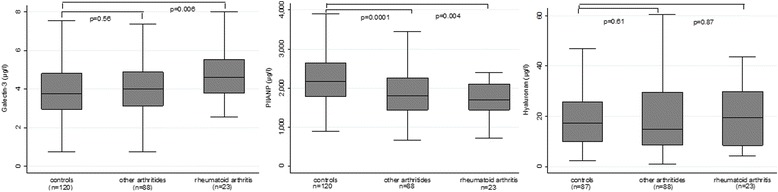
Galectin-3, PIIANP, and hyaluronan levels (median and interquartile range) in pre-RA, non-RA, and healthy subjects. Intergroup comparison between subsets of patients with early, undifferentiated arthritis and healthy controls was carried out using the Mann-Whitney test. *PIIANP* N-terminal propeptide of type IIA collagen, *RA* rheumatoid arthritis

PIIANP was significantly lower in the entire UA group (1800 μg/l, IQR 1439–2252) versus healthy control subjects (2189 μg/l, IQR 1788–2712; *p* < 0.0001; Fig. 2). PIIANP levels did not differ significantly between anti-CCP subsets in the entire UA study population. PIIANP was equally depressed in pre-RA (1704 μg/l, IQR 1439–2105) and non-RA (1801 μg/l, IQR 1436–2260) as compared to healthy controls (*p* < 0.01).

In the univariate analysis, swollen joint count, tender joint count, VAS global, and MRI erosion score emerged as statistically significant. PIIANP correlated inversely with VAS global score (*p* = 0.05) upon multivariate regression in the complete UA study population.

PIIANP correlated positively with MRI erosion score by univariate analysis (*p* = 0.03) in non-RA although this was nonsignificant following adjustment for age and gender (*p* = 0.08). In pre-RA alone, PIIANP correlations with MRI synovitis score, bone marrow edema score, or erosion score did not reach statistical significance.

### Hyaluronan

HYA did not differ between patients with UA (15.1 μg/l, IQR 8.5–29.9) and controls (17.4 μg/l, IQR 10–25.8; *p* = 0.75). Similarly, HYA in pre-RA (*p* = 0.87) and non-RA (*p* = 0.61) did not differ significantly compared to healthy controls. In the entire UA study population and the two subsets, HYA was higher in the anti-CCP negative group compared with the positive group, although this was not statistically significant.

Anti-CCP, CRP, tender joint count, DAS28-CRP, MRI erosion score, MRI bone marrow edema score, and MRI synovitis score emerged as significant in univariate regression analyses with HYA as a dependent variable. HYA correlated inversely with anti-CCP positivity (*p* = 0.02) using multivariate regression.

HYA in the pre-RA subset did not correlate with MRI synovitis score, bone marrow edema score, or erosion score following univariate regression analysis. Conversely, all three MRI scores (synovitis score, *p* = 0.001; erosion score, *p* = 0.18; and bone marrow edema score, *p* = 0.10) emerged as significant by univariate analysis of HYA in non-RA, but did not attain statistical significance after multivariate regression analysis.

Table 3 summarizes the univariate and multivariate analyses in the entire UA study population.

**Table 3:** Univariate and multivariate analyses of galectin-3, PIIANP, and hyaluronan against biochemical, clinical, and imaging findings

	Square root of galectin-3 (*n* = 111)			Log PIIANP (*n* = 111)			Log hyaluronan (*n* = 111)		
Variable	Beta coefficient	95% CI	*p* value	Beta coefficient	95% CI	*p* value	Beta coefficient	95% CI	*p* value
Univariate regression									
Anti-CCP	**–**			**–**			–0.34	–0.87 to 0.18	0.20
C-reactive protein	0.01	0.005 to 0.02	0.002	**–**			0.02	(0.002 to 0.04)	0.05
Swollen Joint count	**–**			–0.08	–0.14 to –0.02	0.006	**–**		
Tender Joint count	**–**			0.009	–0.003 to 0.02	0.15	–0.02	(–0.04 to –0.001)	0.04
VAS – Doctor	**–**			**–**			**–**		
VAS – Pain	0.003	–0.0006 to 0.007	0.10	**–**			**–**		
VAS – Global	**–**			–0.002	–0.005 to 0.0008	0.15	**–**		
DAS28-CRP	**–**			**–**			–0.19	–0.36 to –0.02	0.03
HAQ score	**–**			**–**			**–**		
MRI synovitis score	**–**			**–**			0.11	0.02 to 0.20	0.02
MRI erosion score	**–**			0.04	0.003 to 0.07	0.03	0.05	(–0.01 to 0.11)	0.11
MRI bone marrow edema score	0.02	–0.004 to 0.04	0.11	**–**			**–**		
Multivariate regression									
Anti-CCP	**–**			**–**			–0.59	–1.08 to –0.09	0.02
VAS – Global	**–**			–0.003	–0.007 to –0.00002	0.05	**–**		

Dashes represent nonsignificant results. Results with *p* values ≤0.20 and ≤0.05 were considered significant in univariate and multivariate regression analyses, respectively

*Anti-CCP* antibodies to cyclic citrullinated peptides, *CI* confidence interval, *DAS28-CRP* Disease Activity Score in 28 joints including C-reactive peptide, *HAQ* Health Assessment Questionnaire, *MRI* magnetic resonance imaging, *PIIANP* type IIA collagen N-terminal propeptide, *VAS* visual analogue scale

### Prediction of final diagnosis

**Fig. 3:**
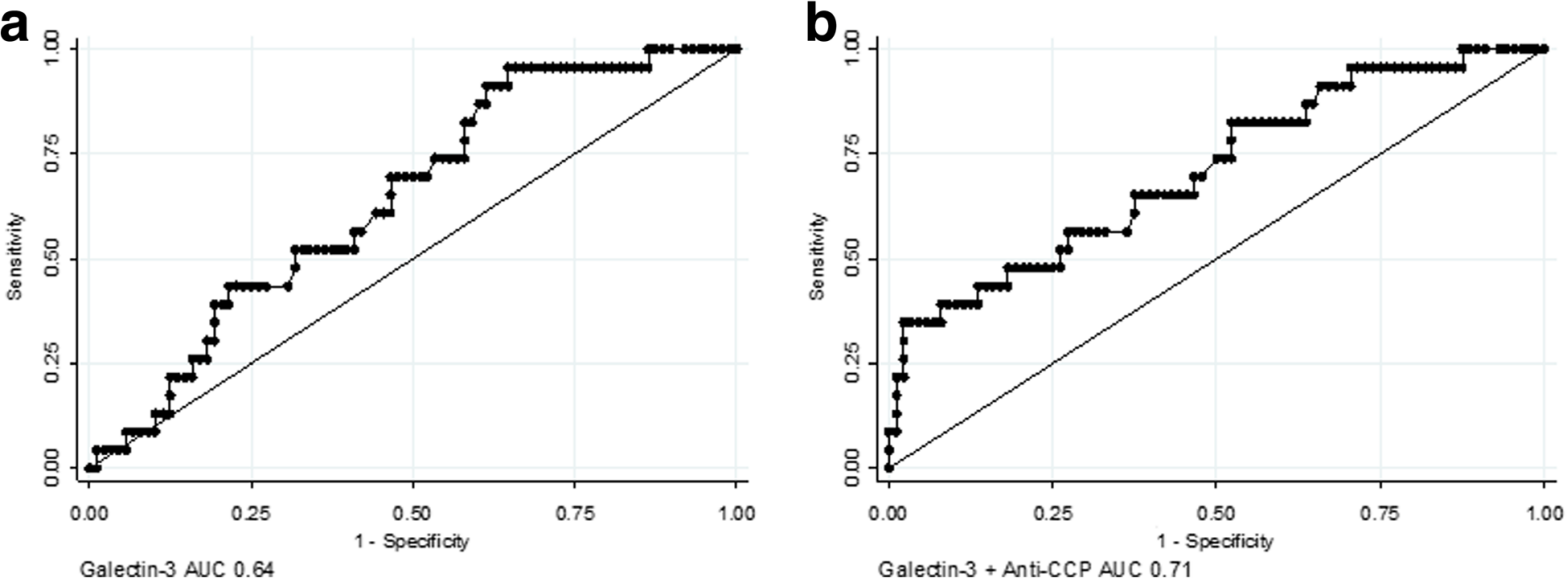
Capability of baseline galectin-3 to predict RA development in patients initially classified with UA. ROC curve analysis was used to assess the ability of galectin-3 alone (**a**) or in combination with anti-CCP (**b**) to distinguish between UA patients who will and those who will not develop RA within 12–23 months follow-up. UA: undifferentiated arthritis.. *Anti-CCP* antibodies to cyclic citrullinated peptides, *AUC* area under curve

The predictive capacity of baseline galectin-3 was studied with logistic regression with adjustment for known independent predictors of developing RA (odds ratio 1.54, *p* = 0.05). Thus one-unit increase in serum galectin-3 increased the odds of developing RA by a factor of 1.54. In addition, the predictive value of baseline galectin-3 regarding future development of RA versus non-RA or healthy controls was also studied using univariate ROC analysis. Univariate area under curve (AUC) for galectin-3 was 0.64 (95% CI 0.53–0.76) for discriminating between pre-RA and non-RA. The corresponding figure for anti-CCP was 0.63 (0.53–0.74), while AUC for the combination of galectin-3 and anti-CCP increased to 0.71 (0.59–0.83; Fig. 3). Similarly, univariate AUC of MRI bone marrow edema score resulted in an AUC of 0.66 (0.54–0.78), while the combination of galectin-3 and bone marrow edema of the wrist yielded an AUC of 0.73 (0.63–0.83), which was not statistically different from galectin-3 + anti-CCP (*p* = 0.78). The AUC of galectin-3 + anti-CCP + bone marrow edema score was 0.74 (0.64–0.85).

## Discussion

Patients presenting with joint complaints of uncertain origin are a common source of concern to clinicians with regard to diagnostic delay and missed therapeutic opportunities. In this first study on galectin-3 and selected seromarkers of joint tissue remodeling in patients with early, undifferentiated arthritis, we report that galectin-3 in serum was increased in pre-RA versus non-RA and a healthy control population. PIIANP, a marker of regenerative cartilage collagen IIA synthesis, was equally depressed in pre- and non-RA, while HYA was in the normal range in either subset. In the entire UA cohort, galectin-3 correlated positively with MRI bone marrow edema score, whereas PIIANP was associated with MRI erosion score and HYA with both MRI synovitis and erosion scores. Galectin-3 discriminated well between pre-RA and non-RA.

The present finding of increased galectin-3 levels in serum of patients with UA who subsequently developed RA as compared with other UA subsets adds to the experimental and clinical evidence that galectin-3 plays a critical role in RA development, e.g., by regulating RA synovial fibroblast functions. Thus, galectin-3 induces distinctive pro-inflammatory cytokine and chemokine expression profiles in RA synovial fibroblasts as compared with dermal fibroblasts [27]. In addition, Ohshima et al. reported that galectin-3 and its binding protein is overexpressed in the RA synovial membrane, particularly at the invading pannus front, and that galectin-3 in the serum and synovial fluid is increased in patients with long-standing RA compared with osteoarthritis and healthy controls [14]. Recently, in a cohort of patients with early, untreated RA we found persistently increased levels of circulating galectin-3 in serum despite subsequent introduction of a targeted synovitis suppressive treatment protocol [15]. Notably, baseline galectin-3 did not correlate with clinical disease activity measures such as tender and swollen joint counts or DAS28-CRP, but correlated positively with MRI erosion and anti-CCP, which is a strong predictor of erosive progression in RA [28]. In the present study on early UA, galectin-3 correlated with CRP but not with joint counts or DAS28-CRP score (Table 3). Galectin-3 also correlated with bone marrow edema, however, reaching statistical significance in the non-RA subset only. This finding may reflect that joint degradative pathways are shared at early stages of different arthritides. Also, the relatively small number of pre-RA patients should be considered. Altogether, these observations concord with evidence from studies using modern imaging technologies that erosive damage in RA may proceed in the absence of clinically detectable synovitis [29–31]. Recently, this has also been reported to take place in joints with persistent bone marrow edema [32]. The abovementioned experimental and clinical studies and the association between increased galectin-3 levels in the serum and bone marrow edema score in UA and early RA independent of current synovitis suggest that galectin-3 may be an important mediator of joint destruction in arthritis.

Galectin-3 expression by RA synovial fibroblasts is stimulated by binding to cartilage oligomeric matrix protein (COMP), a structural cartilage matrix protein belonging to the thrombospondin family [33]. In order to characterize possible links between the increased galectin-3 levels and cartilage and synovial tissue remodeling, we performed additional measurements of serological markers of cartilage collagen regeneration (PIIANP) [16–18] and synovitis-related swellings (hyaluronan) [20]. PIIANP was equally depressed in pre-RA and non-RA and correlated with MRI erosion score in the entire UA population, but not with the occurrence of anti-CCP (Table 3). Previously, we reported that circulating PIIANP was decreased in a large cohort of newly diagnosed, treatment-naive patients with RA classified according to the 1987 criteria, particularly in the anti-CCP subset. In that study, PIIANP did not associate with disease activity, but correlated inversely with anti-CCP titer [18] suggesting a chondrocyte suppressive effect by these autoantibodies [34–36]. More recently, we reported that PIIANP was increased in active, untreated axial spondyloarthritis, particularly in those testing positive for human leukocyte antigen (HLA)-B27, probably reflecting enhanced enthesopathic tissue proliferation in axial joint disease [37]. In the present study on UA, PIIANP was not associated with anti-CCP status, which may be due to the small sample size of anti-CCP-positive patients. However, as in early RA, PIIANP was decreased in the present early UA cohort regardless of the follow-up diagnosis, suggesting that early synovitis of any origin may reduce cartilage regeneration and repair. This is supported by the correlation between decreased baseline PIIANP and MRI erosion score. The inverse correlation between PIIANP and VAS global assessment score probably reflects the link between deep cartilage remodeling and disease activity in UA.

HYA is a well-documented seromarker of synovitis mass in RA [19, 20]. Although the swollen joint count in the entire UA population and the two major subsets was low, wrist MRI synovitis scores ranged between 2–5, 3.5–5.5, and 2–5, respectively. This indicates that a threshold volume of synovial inflammation must be exceeded to be reflected in the circulation and that subclinical synovitis may go unnoticed if tracked by HYA recordings only. Interestingly, HYA correlated inversely with anti-CCP positivity using multivariate regression analysis (Table 3). The unanticipated higher levels of HYA in anti-CCP-negative UA patients may pertain to the report by van Oosterhout et al. that the synovial lining layer is thicker in anti-CCP-negative as compared with anti-CCP-positive RA patients [38].

The ability of galectin-3 to discriminate between pre-RA and non-RA was calculated by ROC analysis (Fig. 2). Galectin-3 alone distinguished fairly well between UA patients who later developed RA and those who did not, with an AUC of 0.64 and, for anti-CCP alone, 0.63. When combining galectin-3 and anti-CCP, AUC increased to 0.71, and with the further addition of bone marrow edema to 0.74. These quite similar figures indicate that galectin-3 may qualify as a future serological marker for prediction of RA development in UA.

Some issues of concern should be addressed. In this study we adopted the 1987 criteria for RA classification [23]. This implies that some individuals would have been categorized as RA according to the 2010 criteria [39], thereby qualifying for exclusion from the study. This potential source of categorization error is underscored by the finding that 7/81 of the non-RA participants tested positive for anti-CCP. On the other hand, this is a study aiming at identifying biologically plausible new seromarkers for clinical use. It was therefore essential to apply these candidate markers to patient subsets diagnosed with optimal specificity. Some patients with UA may only differentiate into RA beyond 12 months [2]. The number of anti-CCP-positive patients was rather small. Accordingly, regression subanalysis with stratification into sero-negatives and sero-positives was not feasible. Smoking status was not available. Major strengths include the collection of a large sample of carefully characterized patients with UA and complete follow-up at 1 year, which allows for separation of patients who did from those who did not develop RA.

## Conclusions

Galectin-3 is increased at the pre-diagnostic stage of RA versus non-RA, and discriminates well between UA patients who will and those who will not develop RA within 12–23 months. UA is associated with a decreased cartilage collagen regenerative response irrespective of synovitis origin.

## Abbreviations

Anti-CCP: Antibodies to cyclic citrullinated peptides
AUC: Area under the curve
CI: Confidence interval
COMP: Cartilage oligomeric matrix protein
CRP: C-reactive protein
DAS28-CRP: Disease Activity Score in 28 joints including C-reactive protein
ELISA: Enzyme-linked immunosorbent assay
ESR: Erythrocyte sedimentation rate
HABP: Hyaluronic acid binding protein
HAQ: Health Assessment Questionnaire
HLA: Human leukocyte antigen
HYA: Hyaluronan
IQR: Interquartile range
MCP: Metacarpophalangeal
MMP: Matrix metalloproteinase
MRI: Magnetic resonance imaging
MTP: Metatarsophalangeal
OA: Osteoarthritis
PIIANP: Type IIA collagen N-terminal propeptide
PIP: Proximal interphalangeal
RA: Rheumatoid arthritis
RAMRIS: Rheumatoid Arthritis Magnetic Resonance Imaging Scoring System
ROC: Receiver operating characteristic
UA: Undifferentiated arthritis
VAS: Visual analogue scale
